# Comparative Analysis of the Conversion of Mandelonitrile and 2-Phenylpropionitrile by a Large Set of Variants Generated from a Nitrilase Originating from *Pseudomonas fluorescens* EBC191

**DOI:** 10.3390/molecules24234232

**Published:** 2019-11-21

**Authors:** Andreas Stolz, Erik Eppinger, Olga Sosedov, Christoph Kiziak

**Affiliations:** 1 Institut für Mikrobiologie, Universität Stuttgart, Allmandring 31, 70569 Stuttgart, Germany; Erik.Eppinger@imb.uni-stuttgart.de (E.E.); Olga.Sosedov@web.de (O.S.); christoph.kiziak@lonza.com (C.K.); 2 BioChem Labor für biologische und chemische Analytik GmbH, Daimlerstr. 5B, 76185 Karlsruhe, Germany; 3 Lonza AG, Rottenstr. 6, 3930 Visp, Switzerland

**Keywords:** nitrilase, reaction specificity, enantioselectivity, structure-function relationship

## Abstract

The arylacetonitrilase from the bacterium *Pseudomonas fluorescens* EBC191 has been intensively studied as a model to understand the molecular basis for the substrate-, reaction-, and enantioselectivity of nitrilases. The nitrilase converts various aromatic and aliphatic nitriles to the corresponding acids and varying amounts of the corresponding amides. The enzyme has been analysed by site-specific mutagenesis and more than 50 different variants have been generated and analysed for the conversion of (*R*,*S*)-mandelonitrile and (*R*,*S*)-2-phenylpropionitrile. These comparative analyses demonstrated that single point mutations are sufficient to generate enzyme variants which hydrolyse (*R*,*S*)-mandelonitrile to (*R*)-mandelic acid with an enantiomeric excess (ee) of 91% or to (*S*)-mandelic acid with an ee-value of 47%. The conversion of (*R*,*S*)-2-phenylpropionitrile by different nitrilase variants resulted in the formation of either (*S*)- or (*R*)-2-phenylpropionic acid with ee-values up to about 80%. Furthermore, the amounts of amides that are produced from (*R*,*S*)-mandelonitrile and (*R*,*S*)-2-phenylpropionitrile could be changed by single point mutations between 2%–94% and <0.2%–73%, respectively. The present study attempted to collect and compare the results obtained during our previous work, and to obtain additional general information about the relationship of the amide forming capacity of nitrilases and the enantiomeric composition of the products.

## 1. Introduction

Nitrilases are hydrolytic enzymes found in many bacteria, fungi, and plants that convert nitriles to the corresponding carboxylic acids and ammonia. They are traditionally assigned according to their substrate preference as aliphatic nitrilases, benzonitrilases, or arylacetonitrilases, although recent results challenge this classification [1,2]. There is a considerable interest in chemistry in the utilization of nitrilases for chemo-, regio- or enantioselective biotransformation reactions [3,4,5,6]. The application of nitrilases for enantioselective reactions has been intensively studied for the production of (*R*)-mandelic acid from (*R*,*S*)-mandelonitrile or benzaldehyde plus cyanide [7]. This reaction is catalysed by different arylacetonitrilases which are often obtained from the species *Alcaligenes faecalis* or organisms which are affiliated to the genus *Pseudomonas* and is already industrially realized in a multi-ton scale [8,9,10,11,12,13,14]. These and other arylacetonitrilases allow in principle also the enantioselective synthesis of other alpha-hydroxycarboxylic acids [15,16,17]. Furthermore, enantioselective reactions have also been observed with arylacetonitrilases carrying various other substituents in the alpha-position (e.g., 2-amino-, 2-halo-, or 2-alkylnitriles) [18,19,20,21].

**Figure 1:**
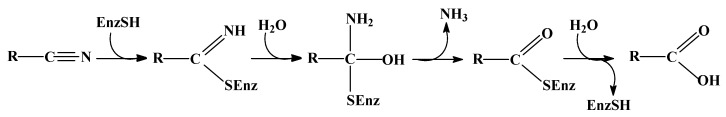
Proposed general reaction mechanism for nitrilases.

Nitrilases possess a catalytical triad, which is composed of a cysteine, a glutamate, and a lysine residue [22]. Moreover, a second glutamate residue seems also to be important for the enzymatic reaction [23]. In the proposed catalytical triad the importance of the cysteine residue has been proven by the detection of a covalently bound reaction intermediate, which was identified as a thioimidate or acylenzyme [24], and by the generation of inactive enzyme variants after the exchange of the relevant cysteine against other amino acid residues [25]. It is therefore generally assumed that the nitriles are initially bound to nitrilases via a cysteine residue in the form of a thioimidate, then hydrated to a tetrahedral intermediate and subsequently deaminated to an acylenzyme, which is finally hydrolysed to the carboxylic acid (Figure 1). It has been proposed that in this reaction the glutamate residue of the catalytical triad acts as general base catalyst and that the lysine stabilizes the tetrahedral intermediate [26].

**Figure 2:**
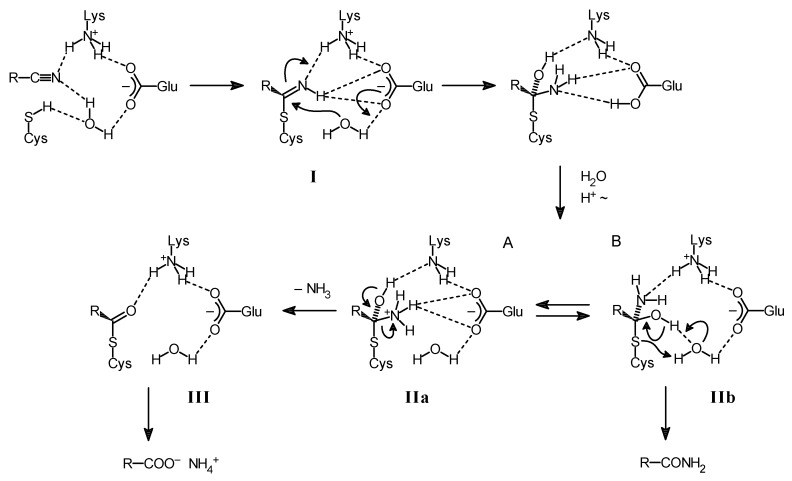
Proposed reaction scheme for the conversion of nitriles to acids or amides by nitrilases [21].

It has been repeatedly reported that some nitrilases form with certain substrates in addition to the acids also the corresponding amides [27,28,29,30,31,32,33]. Therefore, an extended reaction scheme has been suggested to explain this observation (Figure 2) [21,34]. 

This hypothetical reaction scheme suggests that the acids or the amides can be alternatively formed from the tetrahedral intermediate in which the substrate is covalently bound to the catalytically active cysteine residue of the enzyme (IIa, IIb in Figure 2). In the proposed mechanism the formation of the amides formally occurs by a thiol elimination of the active cysteine residue and the acids are formed after the release of ammonia from the tetrahedral intermediate. The release of ammonia (and thus the formation of the acids) would chemically require a protonation of the nitrogen atom originating from the nitrile group in order to obtain a good leaving group. This model gives a plausible explanation for the observation that the amounts of amide formed from a certain substrate strongly vary among different nitrilases and that for a single nitrilase an increased formation of amides can be caused either by the structure of the substrate (e.g., by electron-withdrawing substituents which destabilize the positive charge on the nitrogen atom) or by sterical factors which might hamper the transfer to or the stabilization of the positive charge on the amino group [21].

The explanation of the reaction mechanism and the elucidation of structure/function relationships of nitrilases have been hampered for several years by the inability to crystallize native nitrilases. Presumably, this problem is caused by the peculiar characteristic of enzymatically active nitrilases to form asymmetric spiral structures [35]. To solve these questions, two strategies have been used. Zhang et al. [36] obtained crystals from a C-terminally-deleted variant of the nitrilase from *Synechocystis* sp. PCC6803 and subsequently solved the structure. In a second approach, Sewell et al. used electron microscopy to analyse the structures of different nitrilases [26,37].

## 2. The Nitrilase from *Pseudomonas fluorescens* EBC191

**Figure 3:**
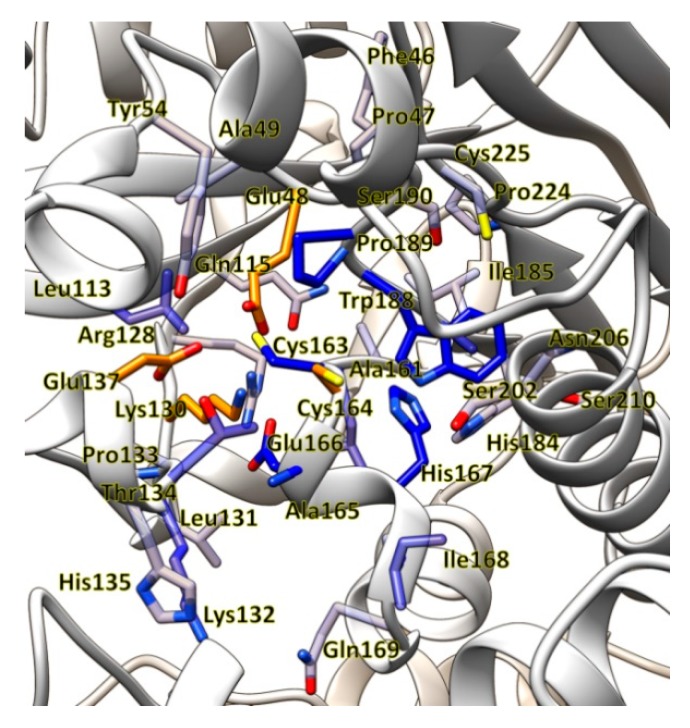
Model for the active centre of the nitrilase from *P. fluorescens* EBC191.

*Pseudomonas fluorescens* EBC191 was isolated after selective enrichment with 2-ethylbenzylcyanide (EBC) as a sole source of nitrogen and shown to synthesize a nitrilase converting a broad range of nitriles [38]. The gene coding for the nitrilase had been cloned and a highly efficient recombinant *E. coli* strain constructed. The amino acid residues involved in catalysis were identified by sequence comparisons with other members of the nitrilase superfamily and mutagenesis [20,23,39] and a model of the active centre generated by homology modelling (Figure 3).

The residues participating in the catalytic triad (Glu48, Lys130, Cys164) and the second catalytically important glutamate residue (Glu137) are shown in orange. Amino acid residues which are located at a distance of less than 10 Å from Cys164 are shown in accordance with their distance to Cys164 in decreasing shades of blue. The model was prepared for the present study based on the structure of the nitrilase from *Synechocystis* sp. PCC6803, PDB 3WUY, using the program Yasara [40]. The resulting homology model was refined by the “md-refine macro” using the default parameters.

**Figure 4:**
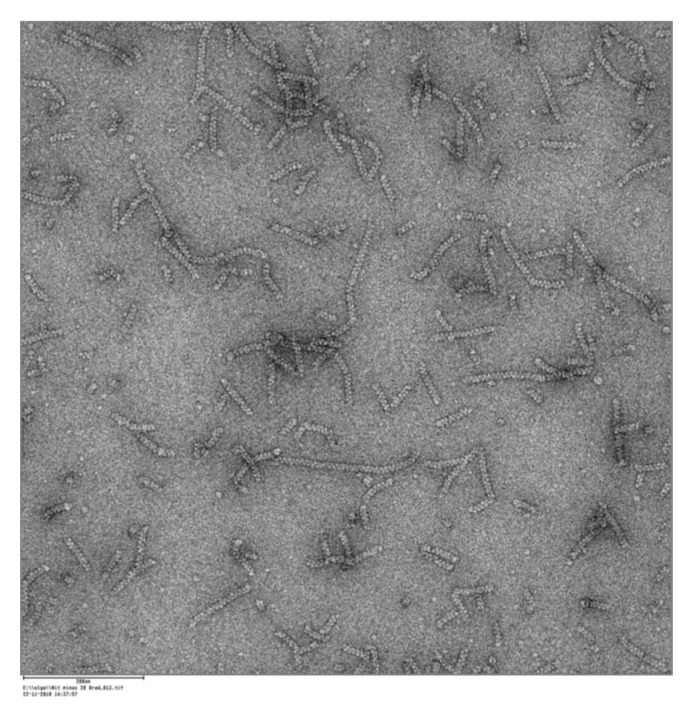
Electron microscopic image of the purified nitrilase from *P. fluorescens* EBC191 [41].

The purified nitrilase from *P. fluorescens* EBC191 forms like other nitrilases spiral structures which can be visualized by electron microscopy (Figure 4).

Therefore, it will be necessary to include the structural constraints caused by the helix formation into the structural model as soon as detailed (cryo) EM data for the nitrilase from *P. fluorescens* EBC191 are available.

**Figure 5:**
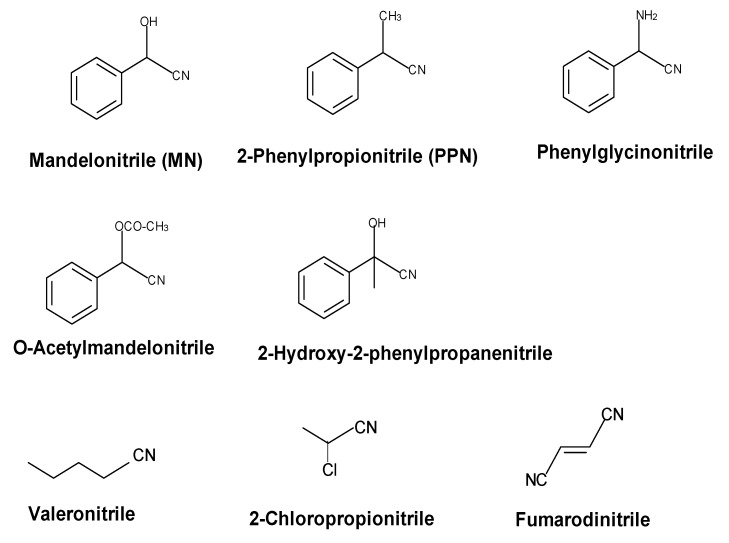
Examples for nitriles which are converted by the nitrilase from *Pseudomonas fluorescens* EBC191.

The wild-type nitrilase hydrolyses various phenylacetonitriles (e.g., mandelonitrile, 2-phenylpropionitrile, phenylglycinonitrile, O-acetylmandelonitrile) and aliphatic mono- and dinitriles to the corresponding carboxylic acids (Figure 5).

In addition to the acids, with some substrates also significant amounts of the corresponding amides are formed [2,20,21,38,42,43,44]. Most of the tested chiral alpha-substituted nitriles were converted by the wild-type nitrilase with a low degree of enantioselectivity. Thus, (*R*,*S*)-mandelonitrile (MN) was hydrolysed preferentially to (*R*)-mandelic acid ((*R*)-MA). In contrast, the nitrilase preferentially formed (*S*)-2-phenylpropionic acid ((*S*)-2-PPA) from (*R*,*S*)-2-phenylpropionitrile ((*R*,*S*)-2-PPN) [20]. The enantioselectivity of the reactions has also been analysed for the hydrolysis of O-acetylmandelonitrile, phenylglycinonitrile (2-aminophenylacetonitrile), 2-chlorophenylacetonitrile, and acetophenone cyanohydrine and it was found that the enzyme formed preferentially the (*R*)-acids from mandelonitrile, O-acetylmandelonitrile, 2-chlorophenylacetonitrile, and acetophenone cyanohydrine. In contrast, the (*S*)-acids were produced in excess during the conversion of (*R*,*S*)-2-PPN and (*R*,*S*)-phenylglycinonitrile [20,21,38,44]. This suggested that electronegative substituents (-OH, -OCOCH_3_, -Cl) induce the preferred formation of the corresponding (*R*)-acids. In contrast, nitriles which carry in the α-position less electronegative or protonable substituents (-CH_3_, -NH_2_) are preferentially converted to the (*S*)-acids. The low degrees of enantioselectivity which were found during the conversion of (*R*,*S*)-MN and (*R*,*S*)-2-PPN (ee-values of 31% for (*R*)-mandelic acid and 65% for (*S*)-2-PPA at about 30% substrate conversion) and the ability of the enzyme to convert—although with a more pronounced enantioselectivity—both enantiomers of O-acetylmandelonitrile proved that the active site of the nitrilase from *P. fluorescens* EBC 191 is in principle able to bind the (*R*)- and (*S*)-enantiomers of α-substituted nitriles (even with rather bulky substituents) [20,38]. The ability of the nitrilase to bind a wide range of substrates with different stereochemistries at the α-position was also substantiated by the observation that the enzyme was able to convert sterically demanding α-,α-disubstituted phenylacetonitriles, such as 2-methyl-2-phenylpropionitrile or 2-hydroxyphenylpropionitrile (acetophenone cyanhydrine), which contain a quaternary carbon atom in the α-position towards the nitrile group [45].

### 2.1. Mutational Studies with the Nitrilase from P. fluorescens EBC191

Several variants of the nitrilase from *P. fluorescens* EBC191 have been generated in order to analyse the factors that determine the substrate-, reaction-, and enantiospecifity of nitrilases. In the course of these investigations, variants have been generated by introducing modifications close to the catalytical active cysteine residue, in the proposed substrate binding site, or by the formation of C-terminally deleted mutants. In addition, experiments were performed in order to increase the amides forming capacity of the enzyme by random and site-directed mutagenesis. Subsequently, these nitrilase variants were used for biotransformation reactions with mandelonitrile and 2-phenylpropionitrile as substrates and analysed for the enantioselectivities of the reactions and amide formation. These two substrates were converted by the purified wild-type enzyme with specific activities of 33 and 4.1 U/mg of protein, respectively [23,39,46,47,48]. Mandelonitrile and 2-phenylpropionitrile were used as model substrates, because they were converted by the wild-type enzyme with a different stereopreference (see above). These compounds show in aqueous solutions at neutral pH-values a rather different behaviour. While 2-phenylpropionitrile is completely stable under these conditions, mandelonitrile exists in equilibrium with benzaldehyde and HCN. This results in a pronounced tendency of mandelonitrile to racemize in neutral or alkaline aqueous solutions which leads in the presence of an enantioselective nitrilase to a dynamic kinetic resolution. This is industrially exploited for the almost stoichiometrical conversion of benzaldehyde plus cyanide to (*R*)-mandelic acid [8,9,12].

The analysis of the large set of nitrilase variants generated throughout the years, showed that the catalytical properties of the enzyme could be significantly modified by single point mutations [2,23,39,45,46,47,48]. Thus, enzyme variants were found which hydrolysed (*R*,*S*)-mandelonitrile preferentially either to (*R*)- or (*S*)-mandelic acid [23,39,46,47,48]. In addition, nitrilase variants were identified which either formed almost exclusively mandelic acid or mandelamide [23,46,47]. In the present study, it was attempted to perform a comparative analysis of the variants to give an overview about the catalytical potential of a single nitrilase and its variants and to obtain additional information which might allow a better understanding of the features which govern the reaction- and enantiospecificity of nitrilases.

### 2.2. Mutations Which Result in the Increased Formation of (R)-Acids

**Figure 6:**
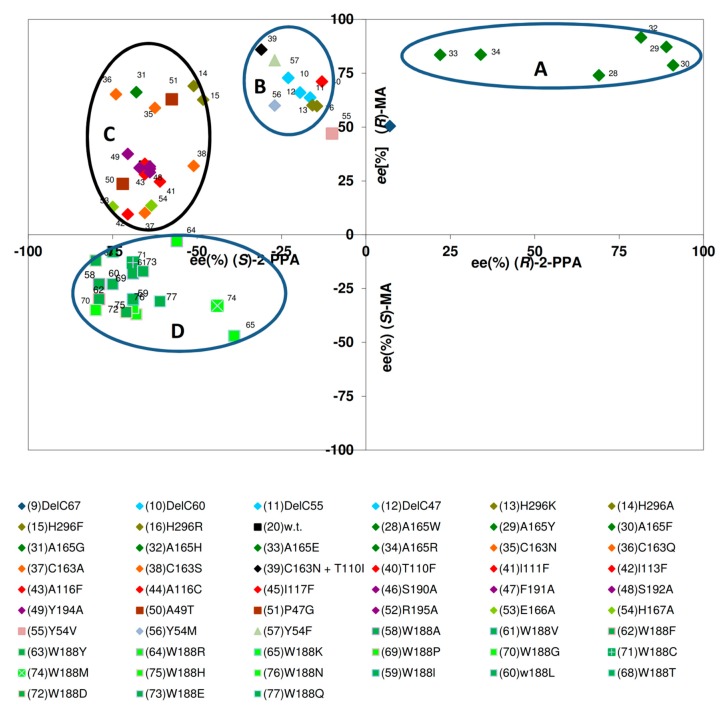
Correlation of the enantiomeric composition of the acids formed from (*R*,*S*)-mandelonitrile and (*R*,*S*)-2-phenylpropionitrile by different nitrilase variants. The indicated values were obtained from the experimental data presented in the articles of Kiziak et al. (2007) [39], Kiziak and Stolz (2009) [46], Sosedov et al. (2010) [47], Baum et al. (2012) [45], Sosedov and Stolz (2015) [23], and includes also some unpublished results. The respective reaction mixtures contained initially 10 mM of (*R*,*S*)-mandelonitrile or (*R*,*S*)-2-PPN. The ee-values were determined after about 30% substrate turn-over.

In order to compare the influence of different mutations on the enantiomeric composition of the formed acids, the results were analysed that had been obtained previously for the conversion of (*R*,*S*)-MN and (*R*,*S*)-2-PPN. Therefore, for each enzyme variant, the ee-values that were obtained during the conversion of (*R*,*S*)-mandelonitrile were plotted against those values that were observed during the conversion of (*R*,*S*)-2-PPN (each after about 30% substrate conversion). These comparisons established that by single point mutations nitrilase variants could be generated which formed either (*R*)-mandelic acid with ee-values up to 91% or (*S*)-mandelic acid with an ee-value of 47%. Similarly, during the conversion of (*R*,*S*)-2-PPN, ee-values between 80% for (*S*)-2-PPA and 91% for (*R*)-2-PPA were observed (Figure 6).

The comparisons suggested that four groups of nitrilase variants could be defined. One group encompasses nitrilase variants which form (*R*)-MA and (*R*)-2-PPA with extraordinary high enantioselectivities (Figure 6, group A). These comparisons confirmed that only those nitrilase variants that formed (*R*)-mandelic acid with ee-values >75% formed preferentially (*R*)-2-PPA (Figure 6). This attribute inevitably correlated with the presence of comparably large amino acid residue in the amino acid residue 165 in the direct neighbourhood of the catalytical active cysteine residue (towards the C-terminus). This resulted in the generation of enzyme variants that formed (*R*)-mandelic acid with ee-values >90% (compared to an ee-value of about 31% for the wild-type enzyme under the same conditions).

The observed interdependence of a pronounced selectivity for the formation of (*R*)-MA from (*R*,*S*)-MN and the presence of a large amino acid residue in direct neighbourhood to the catalytical active cysteine residue nicely correlates to results obtained with other nitrilases. Thus, in the nitrilase from *Alcaligenes faecalis* ATCC8750 (which forms (*R*)-MA with a high enantiomeric surplus [8,9]) a tryptophan residue is present at the homologous position.

The second group of nitrilase variants (Figure 6, group B) showed compared to the wild-type enzyme a significantly decreased enantioselectivity for the formation of (*S*)-2-PPA and a (slight) tendency for an increased formation of (*R*)-mandelic acid. In this group mainly variants cluster which carry mutations in the C-terminal region or in Tyr 54.

The largest group of variants (Figure 6, group C) behaved similar to the wild-type enzyme and hydrolysed (*R*,*S*)-mandelonitrile with a low degree of enantioselectivity preferentially to (*R*)-mandelic acid and (*R*,*S*)-2-PPN preferentially to (*S*)-2-PPA. These variants showed only a low degree of variance regarding the formation of (*S*)-2-PPA (ee-values of about 50%–80%), but more pronounced differences for the formation of (*R*)-MA (ee-values of about 10%–70%).

The nitrilase variants belonging to these three groups generally exhibited with (*R*,*S*)-mandelonitrile a more pronounced tendency towards the formation of the (*R*)-acids than with (*R*,*S*)-2-PPN. Thus, almost all variants belonging to the group A, produced (*R*)-MA with a higher ee-value than (*R*)-2-PPA. This effect was even more pronounced for the variants clustering in groups B and C which formed preferentially (*R*)-MA, but (*S*)-2-PPA (Figure 6).

### 2.3. Mutations Which Result in the Increased Formation of (S)-Mandelic Acid

In the course of the mutational studies also a group of variants had been generated which converted (*R*,*S*)-MN preferentially to (*S*)-MA (Figure 6, group D). In all these variants, the tryptophan residue Trp188 was exchanged against smaller amino acid residue. This group of variants differed from the other enzyme variants also in other aspects and most of these enzymes formed from (*R*,*S*)-mandelonitrile more mandelamide than mandelic acid (see below).

A bulky tryptophan residue at the position corresponding to Trp188 seems to be highly conserved among nitrilases and seems generally to be required to impede the binding of (*S*)-mandelonitrile (or promote the binding of (*R*)-mandelonitrile). This can also be deduced from a previous study by Robertson et al. (2004) [19] who described the existence of nitrilases that show some preference for the formation of (*S*)-mandelic acid from (*R*,*S*)-mandelonitrile. In this study it was shown that among 48 different mandelonitrile hydrolyzing nitrilases obtained by a metagenomic approach only four were (*S*)-selective and formed (*S*)-mandelic acid with ee-values up to 30%. Sequence alignments constituted evidence that in these nitrilases the usually highly conserved relevant tryptophan residue is replaced by a tyrosine or a threonine residue [23].

### 2.4. Correlation Between the Relative Proportions of Amides Formed from (R,S)-Mandelonitrile or (R,S)-2-Phenylpropionitrile

Previously, it had been described that different nitrilases demonstrate various degrees of amide forming capacity and it was found for the nitrilase from *P. fluorescens* EBC 191 and also for a nitrilase from the plant *Arabidopsis thaliana* that in general larger amounts of amides were formed from nitriles that carried in the α-position electronegative substituents, e.g., halide- or hydroxy-groups [21,31].

**Figure 7:**
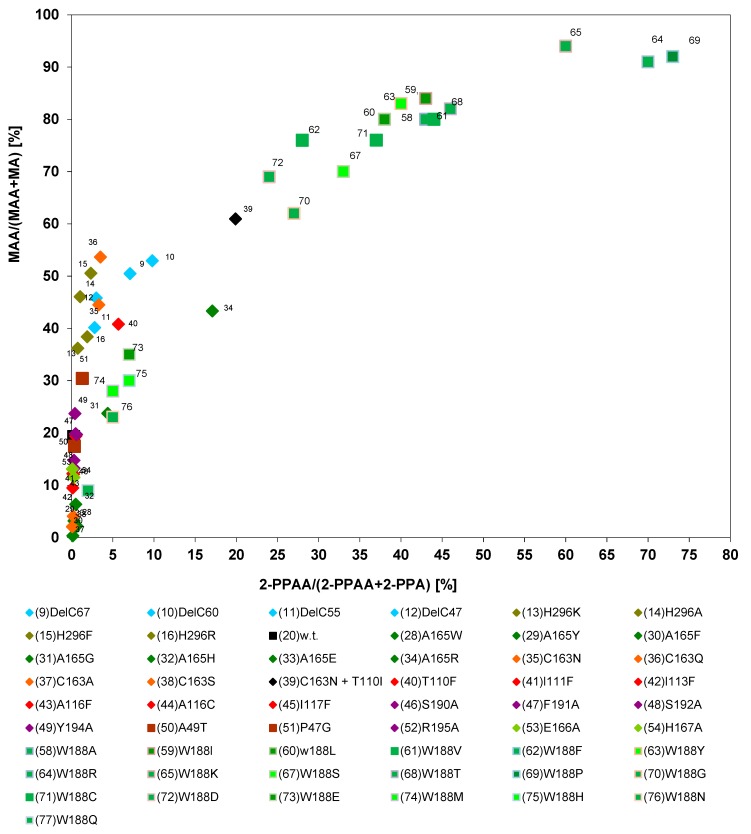
Correlation between the amounts of amides formed from (*R*,*S*)-mandelonitrile and (*R*,*S*)-2-phenylpropionitrile by different nitrilase variants. The data were taken from previous publications (see Figure 6).

The wild-type nitrilase from *P. fluorescens* EBC191 formed from (*R*,*S*)-MN about 17% mandelamide (MAA), but from (*R*,*S*)-2-PPN only about 0.2% of the corresponding amide 2-phenylpropionamide (2-PPAA) [20]. To further analyse the correlation between amide formation from (*R*,*S*)-MN and (*R*,*S*)-2-PPN, the available variants of the nitrilase from *P. fluorescens* EBC191 were compared for the respective reactions. This proved that by single point mutations the amounts of mandelamide formed from (*R*,*S*)-MN could be changed between <1% and 94%. The respective values for the conversion of (*R*,*S*)-2-PPN varied between <0.1% and 73% 2-PPAA (Figure 7). The individual enzyme variants formed generally more mandelamide from (*R*,*S*)-mandelonitrile than 2-PPAA from (*R*,*S*)-2-PPN. This confirmed the assumption that electron-withdrawing substituents (as the hydroxyl-group in mandelonitrile) induce an increased formation of amides.

Almost all enzyme variants that produced between 0%–20% mandelamide from (*R*,*S*)-mandelonitrile resembled the wild-type enzyme and formed only rather low amounts (<1%) of 2-PPAA from (*R*,*S*)-2-PPN (Figure 7). The comparison of the individual variants also showed that in those variants which formed more than 50% of mandelamide from (*R*,*S*)-mandelonitrile a significant change between the degrees of amide formation can occur. Thus, the wild-type nitrilase of *P. fluorescens* formed about 17% of mandelamide from (*R*,*S*)-mandelonitrile, but only about 0.2% of 2-PPAA from (*R*,*S*)-2-PPN. This corresponds to an 85-fold higher rate for the formation of mandelamide than 2-PPAA. The most efficient amide forming mutant derived from this enzyme by a single mutagenic event (Trp188Lys) formed 94% of mandelamide from (*R*,*S*)-mandelonitrile and about 60% of 2-PPAA from (*R*,*S*)-2-PPN which corresponds only to a 1.6-fold higher rate for the formation of mandelamide compared to 2-PPAA.

Several types of mutations could be identified which resulted in an increased tendency of the variants to form amides (Figure 7). The most pronounced increases in amide formation were observed when the tryptophan residue Trp188 was exchanged against a smaller residue and it was found that three of these mutants (Trp188Arg, Trp188Lys, Trp188Pro) formed more than 90% of mandelamide from (*R*,*S*)-MN groups [23]. An increased formation of mandelamide was also found with nitrilase variants that carried deletions of approximately 40–60 amino acids at the C-terminus or in the histidine residue His296, which is located close to the C-terminus. (The full-length enzyme consists of 350 amino acid residues.) Up to 50% of mandelamide were also formed by variants which possessed NH*_2_*-functions (as present in asparagine, glutamine, or arginine) close to the catalytical centre (e.g., the enzyme variants Cys163Gln or Cys163Asn) [39,46]. Furthermore, also the exchange of a threonine residue in position 110 against an isoleucine or phenylalanine or the replacement of an asparagine residue in position 206 by isoleucine-, lysine-, tyrosine-, or aspartate-residues increased the amide formation [39,46,47]. In addition, also the presence of a tyrosine residue in position 54 (as present in the wild-type nitrilase from *P. fluorescens* EBC191) results in a stronger tendency to form amides compared to the presence of almost all other possible amino acids at this position [45].

### 2.5. Correlation Between the Relative Amounts of Amides Formed from (R,S)-Mandelonitrile or (R,S)-2-Phenylpropionitrile and the Enantiomeric Composition of the Amides

The wild-type nitrilase from *P. fluorescens* converted (*R*,*S*)-mandelonitrile and (*R*,*S*)-2-PPN each to opposite enantiomers of the respective acids and amides. Thus, (*R*)-mandelic acid and (*S*)-mandelamide and (*S*)-2-PPA and (*R*)-2-PPAA were preferentially formed from the racemic nitriles [20,21]. Furthermore, it was observed that the nitrilase transformed the two enantiomers of mandelonitrile (and also of O-acetylmandelonitrile) to considerably different amounts of the corresponding amides as side-products. Thus, (*S*)-mandelonitrile was converted to almost equimolar concentrations of (*S*)-mandelamide and (*S*)-mandelic acid, but (*R*)-mandelonitrile was transformed to (*R*)-mandelic acid and (*R*)-mandelamide in a ratio of about 85:15 [21,43,44].

**Figure 8:**
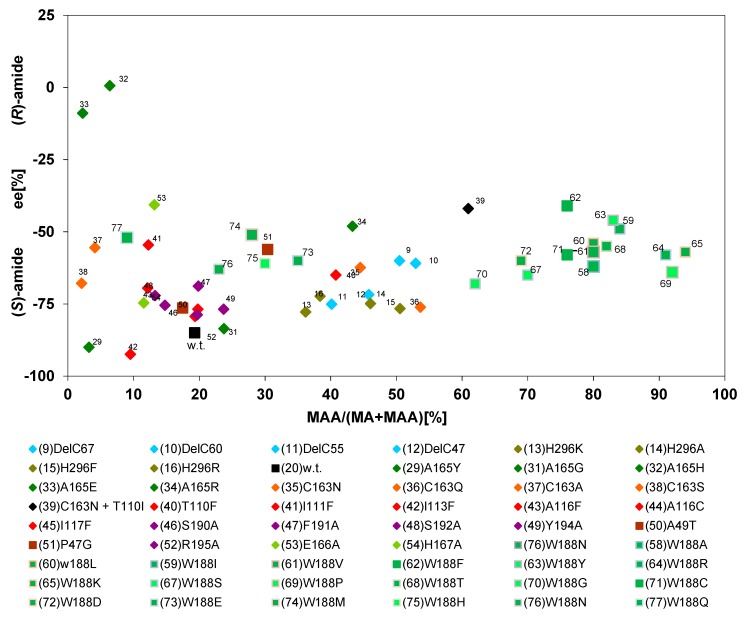
Relationship between the amounts of mandelamide formed from (*R*,*S*)-mandelonitrile and the enantiomeric composition of the formed amide. The data were taken from previous publications (see Figure 6). In order to exclude errors from inaccurate measurements only those reactions were analysed in which more than 2% of mandelamide were formed from (*R*,*S*)-mandelonitrile.

**Figure 9:**
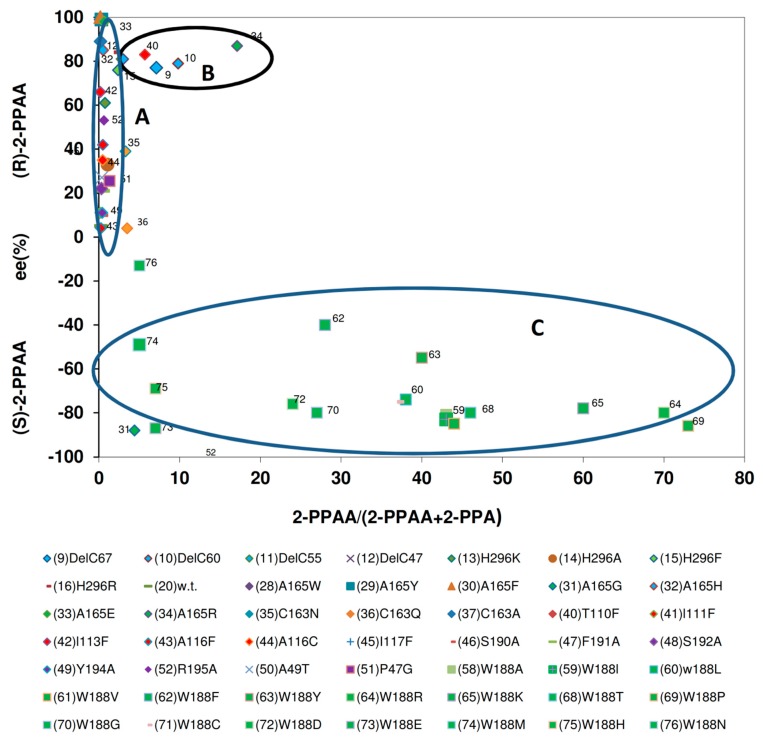
Relationship between the amounts of 2-phenylpropionamide formed from (*R*,*S*)-2-phenylpropionitrile and the enantiomeric composition of the amide. The data were taken from previous publications (see Figure 6).

To gain additional information, two plots were produced which related the relative amounts of amides formed with the enantiomeric composition of the amides (Figure 8 and Figure 9). The graphical representation of the data for the formation of mandelamide from (*R*,*S*)-MN demonstrated that the enantiomeric composition of the amides was largely independent from the relative amounts of amides formed. Thus, variants were compared which produced between 2%–94% of mandelamide. Nevertheless, almost consistently (*S*)-mandelamide was preferentially formed with ee-values of 40%–85% (Figure 8). These experiments established that the mutations resulting in the formation of increased amounts of mandelamide from (*R*,*S*)-mandelonitrile did only marginally influence the enantioselectivity.

In contrast to the conversion of mandelonitrile by the variants, significant differences in the enantiomeric composition of the products formed were observed when the formation of 2-PPAA from (*R*,*S*)-2-PPN was compared. Thus, those variants which formed as the wild-type enzyme only minor amounts of 2-PPAA (<3%) preferentially synthesized (*R*)-2-PPAA with different degrees of enantioselectivity (Figure 9, group A).

The second group (Figure 9, group B) encompassed variants which formed 3%–17% of 2-PPAA and showed compared to the wild-type enzyme a slightly enhanced enantioselectivity for the formation of (*R*)-2-PPAA with ee-values of about 80%.

The third group (Figure 9, group C) assembled those variants in which the tryptophan residue in position 188 had been replaced by a smaller amino acid residue. These variants all formed preferentially (*S*)-2-PPAA. Furthermore, it was found that the large majority of these variants synthesized (*S*)-2-PPAA with ee-values of about 80%, but that the amounts of 2-PPAA formed differed largely among the variants.

### 2.6. Comparison of the Enantiomeric Composition of the Acids and Amides Formed During the Turn-Over of (R,S)-Mandelonitrile or (R,S)-2Phenylpropionitrile

**Figure 10:**
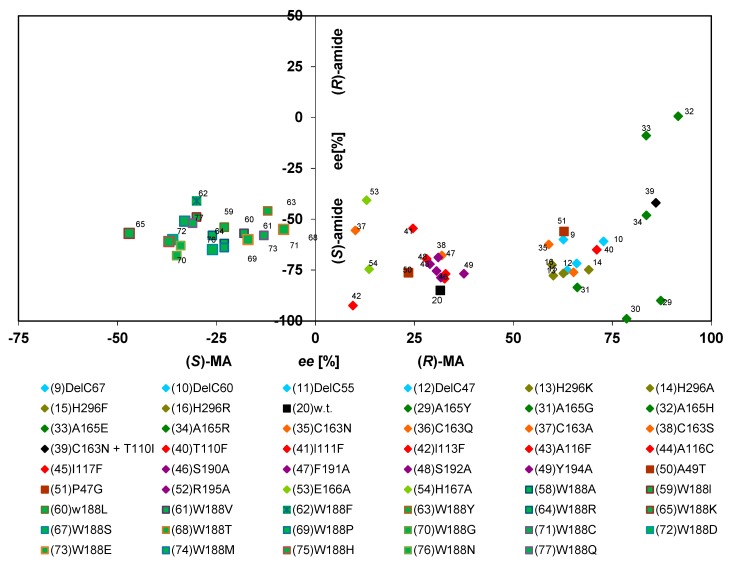
Correlation of the enantiomeric composition of the amides and acids formed from (*R*,*S*)-mandelonitrile by various nitrilase variants. The data were taken from previous publications (see Figure 6). But those variants were excluded from the analysis which formed less than 2% of mandelamide (in relation to the amounts of mandelic acid formed).

**Figure 11:**
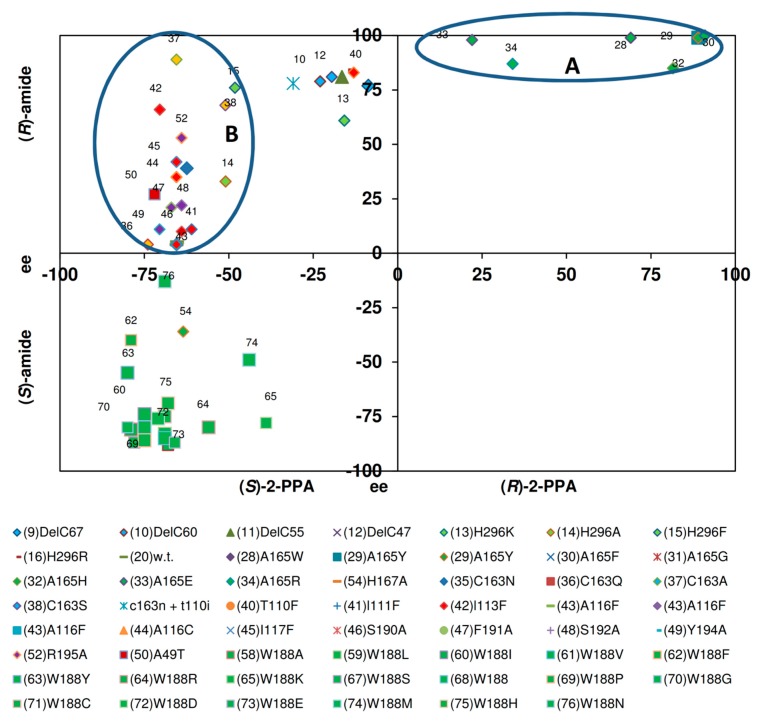
Correlation of the ee-values of the amides and acids formed from (*R*,*S*)-2-phenylpropionitrile by various nitrilase variant. The data were taken from previous publications (see Figure 6), but those variants were excluded from the analysis which formed less than 1% of 2-PPAA (in relation to the amounts of 2-PPA formed).

As indicated above, the wild-type nitrilase from *P. fluorescens* EBC191 preferentially converted (*R*,*S*)-mandelonitrile to (*R*)-mandelic acid and (*S*)-mandelamide and (*R*,*S*)-2-PPN to (*S*)-2-PPA and (*R*)-2-PPAA [20,21]. In the following, it was analysed if this was a general trait of the available nitrilase variants. Therefore, for the nitrilase variants the enantiomeric excesses of the acids and amides produced were correlated to each other (Figure 10 and Figure 11).

The plot for the turn-over of (*R*,*S*)-mandelonitrile indicated that the acids and amides forming reactions did not show any direct correlation in their enantioselectivities. Almost all enzyme variants that formed (*R*)-mandelic acid with ee-value of 10%–90% or (*S*)-mandelic acid with ee-values up to 50% formed (*S*)-mandelamide with ee-values between 40%–95%, but without any recognizable correlation between the enantioselectivities of both reactions (Figure 10). The only exception from this rule were two mutants (Ala165His and Ala165Glu) which exhibited an increase in the enantioselectivity towards the formation of (*R*)-mandelic acid in combination with a slightly decreased formation of (*S*)-mandelamide (Figure 10).

The corresponding plot for the conversion of 2-PPN indicated that also with this substrate the enantiomeric composition of the formed amide was to a large extent independent of the enantioselectivity of the acid formation. Furthermore, the plot suggested that the variants could be separated into two groups (Figure 11).

One group (group A) consisted of the variants which preferentially formed (*R)*-2-PPA and (*R*)-2-PPAA with a high degree of selectivity. These variants formed (*R*)-2-PPAA with ee-values >75%. The second group (group B) of variants (which also encompassed the wild-type enzyme) formed (*S*)-2-PPA with ee-values of about 50%–75%, but formed (*S*)- or (*R*)-(2)-PPAA with ee-values varying between about 80% for (*S*)- or (*R*)-2-PPAA. Thus, it appears that with (*R*,*S*)-2-PPN the enantiopreference for the formation of the amide could be easily modified by mutations and that the enantiopreference for the formation of the acid is rather conserved. This is in contrast to the situation observed during the conversion of (*R*,*S*)-mandelonitrile (see Figure 10).

### 2.7. Comparison of the Relative Amounts of Mandelamide Formed and the Enantiomeric Excess of the Mandelic Acid Produced During the Turn-Over of (R,S)-Mandelonitrile

**Figure 12:**
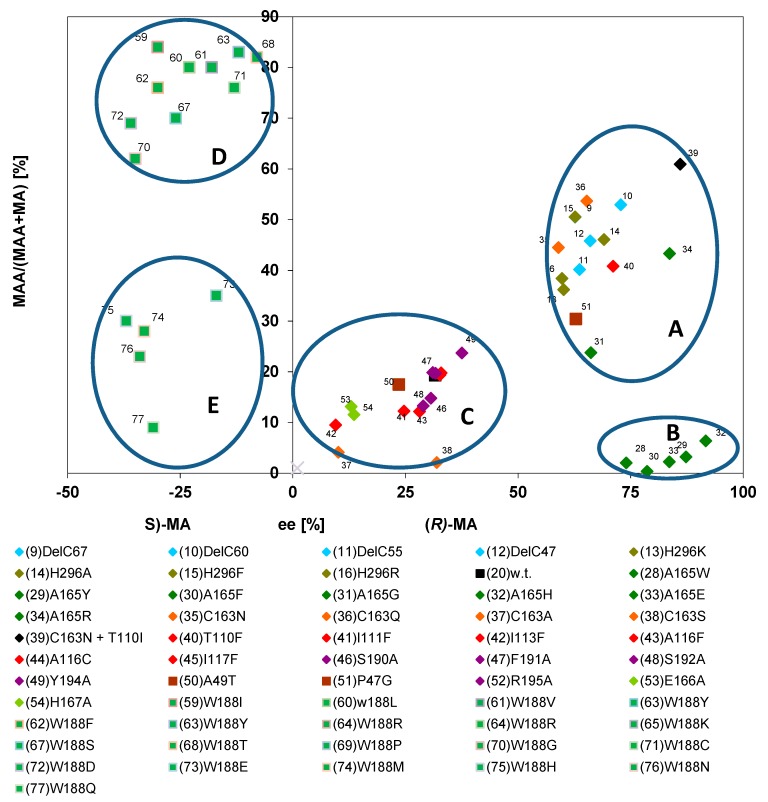
Correlation between the relative amounts of mandelamide formed and the ee-values of the mandelic acid produced during the conversion of (*R*,*S*)-mandelonitrile. The data were taken from previous publications (see Figure 6).

The wild-type nitrilases from *P. fluorescens* EBC191 and *A. faecalis* ATCC8750 demonstrated with (*R*,*S*)-mandelonitrile as a substrate very clear differences with respect to enantioselectivity and amide formation. Thus, the nitrilase from *A. faecalis* showed a high degree of enantioselectivity for the formation of (*R*)-mandelic acid and produced almost no mandelamide. In contrast, for the nitrilase from *P. fluorescens* a low degree of enantioselectivity and a pronounced formation of mandelamide were reported [39]. It was therefore analysed if this correlation was also valid for the different nitrilase variants from *P. fluorescens* EBC191. Therefore, a diagram was generated which correlated the relative amounts of mandelamide with the enantiomeric composition of (*R*)-mandelic acid formed. Surprisingly, this representation of the data suggested that for most enzyme variants increased enantioselectivity for the formation of (*R*)-mandelic acid correlated with an increase in the relative amounts of the produced mandelamide (Figure 12, group A). In addition, a small group of enzyme variants was identified (Figure 12, group B) which were highly enantioselective for the formation of (*R*)-mandelic acid, but which formed only very low amounts of mandelamide. The nitrilase from *A. faecalis* is the “archetypical” representative of this small group of nitrilases. These variants all carry (such as the nitrilase from *A. faecalis*) a large substituent in direct neighbourhood to the catalytical active cysteine residue and were already identified in the plot shown in Figure 6 because of their extraordinary enantioselectivity for the formation of (*R*)-mandelic acid.

The graphic also suggested that the variants carrying an exchange in Trp188 (and thus forming preferentially (*S*)-mandelic acid and (*S*)-mandelamide) could be separated into two groups with different tendencies to form mandelamide. Thus, many of the variants carrying polar amino acids in position 188 (e.g., Glu, Gln, Asn, His) synthesized only low amounts of mandelamide (Figure 12, group E) compared to all other Trp188X variants (Figure 12, group D).

### 2.8. Comparison of the Reaction- and Enantiospecificity of the Nitrilase Variants During the Conversion of (R,S)-Mandelonitrile

The nitrilase variants converted (*R*,*S*)-mandelonitrile to different amounts of (*R*)- or (*S*)-mandelic acid and (*R*)- or (*S*)-mandelamide. In the course of our investigations no indications for any enantioconversion were observed during the formation of the acids or amides. Therefore, for the final comparisons it was attempted to define a parameter, which reflected the total enantiomeric composition of the products formed in a single numerical term. The concentrations of the (*S*)-enantiomers of mandelic acid and mandelamide at the relevant point of conversion (about 30% substrate turn-over, see above) were added up and the respective (*R*)-enantiomers were treated in the same way, as exemplified in Table 1. These values should in principle reflect the enantiodiscrimination of the nitrilase variants during the initial binding of the nitriles.

**Figure 13:**
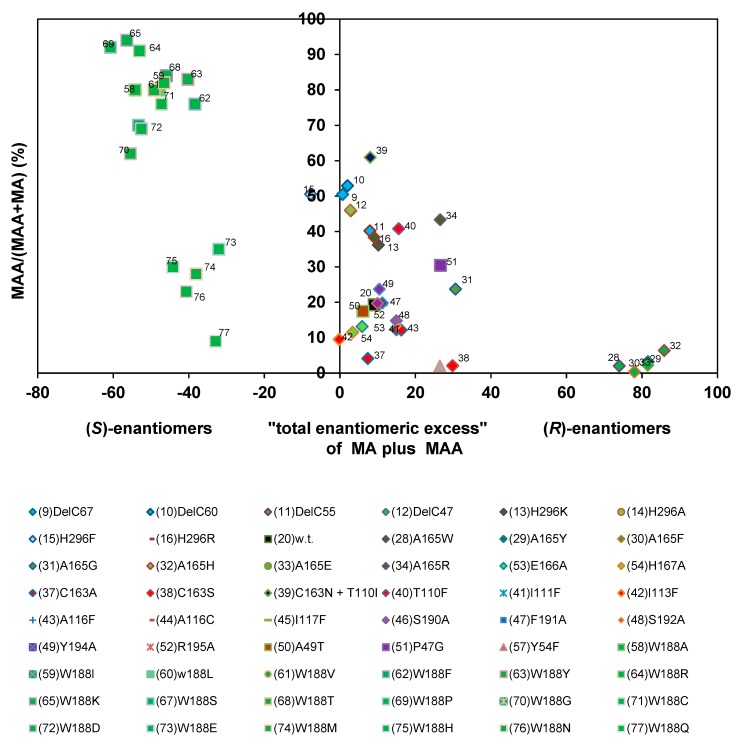
Correlation of the relative amounts of mandelamide formed and the “total enantiomeric excess” of the products formed during the turn-over of (*R*,*S*)-mandelonitrile. The data were taken from previous publications (see Figure 6) and the calculations performed as described in the text and exemplified in Table 1.

Subsequently, the relative amounts of mandelamide formed from (*R*,*S*)-mandelonitrile were plotted against the enantiomeric excesses of the products (=sum of the concentrations of the (*S*)-enantiomers subtracted from the sum of the concentrations of the (*R*)-enantiomers divided by the sum of the concentrations of the (*S*)- plus (*R*)-enantiomers). This type of plot confirmed the results that the nitrilase variants could be differentiated in three major groups (Figure 13).

The comparisons shown in Figure 12 and Figure 13 established that the integration of the enantiomeric composition of the amides into the calculations resulted for most enzyme variants (those combined in group A in Figure 12) in significantly lower values for the (*R*)-selectivity. This was caused by the fact that for these enzyme variants the increased ee-values for (*R*)-mandelic acid correlated with an increased amide formation and that the preferentially formed enantiomer of the amide possessed an opposite stereochemistry compared to the acid. This resulted in the chosen graphical presentation of the data in the clustering of most enzyme variants in a group of enzymes which exhibited *in-summa* during the turn-over of (*R*,*S*)-mandelonitrile only a low preference for the formation of the (*R*)-enantiomers of the two relevant products (acid and amide). In most of these enzyme variants the alanine residue in position 165 of the nitrilase from *P. fluorescens* was conserved (with the exceptions of Nit(A165G) and Nit(A165R)). Thus, it appears that this group of enzymes accepts (*S*)- and (*R*)-mandelonitrile as substrates without a significant preference for one of the substrate enantiomers. In combination with the observations that these variants preferentially form the (*R*)-acid together with the (*S*)-amide and the previous demonstration that nitrilases intermediately form a covalent adduct between the C-atom originating from the nitrile moiety and the catalytical active cysteine residue (Figure 1) this suggests that there is a difference in the probability of the way how the tetrahedral intermediate is resolved. Thus, it appears that the enzyme bound (*R*)-form can be easier deaminated than the enzyme bound (*S*)-form. This results finally in the observed preference for the formation of (*R*)-mandelic acid and (*S*)-mandelamide. The graphical presentation shown in Figure 13 did not significantly change the position of those enzymes that were already identified in Figure 12 as outstanding (groups B and C), because the members of these groups combined the enantioselective formation of (*R*)-mandelic acid with a rather low degree of amide formation or formed (S)-mandelic acid plus (*S*)-mandelamide.

The group of enzymes, which combined the highly enantioselective formation of (*R*)-mandelic acid with a low degree of amide formation (group B in Figure 12) comprised the enzyme variants Nit(A165W), Nit(A165Y), Nit(A165F), Nit(A165H), and Nit(A165E). These enzymes possessed aromatic amino acids or other bulky substituents in the position, which is (C-terminally) in direct neighbourhood to the catalytical active cysteine residue. This type of substituents occurs to accomplish two types of functions, an enhancement in enantioselectivity and a significant reduction in amide formation. Furthermore, it can be deduced that this type of enzyme binds (*R*)-mandelonitrile with a pronounced preference and that the binding or conversion of (*S*)-mandelonitrile is impeded by the presence of a bulky substituent in direct neighbourhood to the catalytically active cysteine residue. This could also explain the increased amounts of the (*R*)-enantiomers of the amide formed and the decreased amounts of the totally formed amides (as the lower amounts of enzyme bound (*S*)-enantiomers will also decrease amide formation because of the reduced tendency of the enzyme to convert (*R*)-mandelonitrile to (*R*)-mandelamide). The group of variants carrying a Trp188X mutation was also in the plot shown in Figure 13 clearly separated from all other variants (group C). Furthermore, a comparison of Figure 12 with Figure 13 proved that these variants indeed were the only group that clearly showed a preference for the conversion of (*S*)-mandelonitrile, as both the (*S*)-enantiomers of mandelamide and mandelic acid were formed.

### 2.9. Comparison of the Reaction- and Enantiospecificity of the Nitrilase Variants During the Conversion of (R,S)-2-Phenylpropionitrile

In the previous paragraph, the conversion of mandelonitrile by the enzyme variants was analysed. In the following, it was attempted to perform the same comparisons for the turn-over of (*R*,*S*)-2-PPN. The interpretation of the relevant data obtained during the conversion of (*R*,*S*)-2-PPN was much more difficult than the previous analysis because with 2-PPN as substrate in general much lower amounts of amides were formed. This complicated especially the chiral analysis and thus the interpretation of the experimental results. Furthermore, the nitrilase variants differed much stronger in the enantioselectivity of 2-PPA-formation than observed for the formation of mandelic acid from (*R*,*S*)-mandelonitrile.

**Figure 14:**
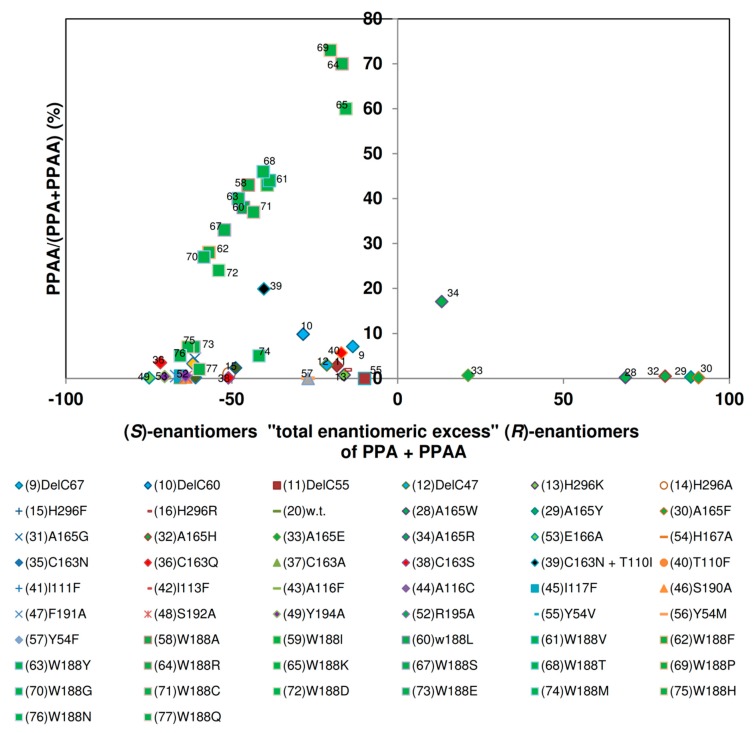
Correlation between the relative amounts of 2-phenylpropionamide formed and the “total enantiomeric excess” of the products formed during the conversion of (*R*,*S*)-2-phenylpropionitrile. The data were taken from the previous publications (see Figure 6).

The investigated nitrilase variants could be differentiated in four groups in respect to the relationship of the “total enantiomeric excess” and the amounts of 2-PPAA formed (Figure 14). The largest group of variants exhibited similar “total enantiomeric excesses” for the formation of the (*S*)-enantiomers as the wild-type nitrilase from *P. fluorescens* EBC191 (ee-values of about 50%–80%) and formed only low amounts of 2-PPAA. The enzymes of the second group showed almost no enantioselectivity. Members of this group were *i.a*. the four C-terminally deleted enzyme variants studied, the variants carrying the point mutations Nit(His296Lys) und Nit(His296Arg) within the C-terminus and a mutant carrying an exchange in the amino acid residue threonine- 110 (Nit(Thr110Phe)).

In the third group all enzyme variants were combined that showed a strong bias for the formation of (*R*)-2-PPA and formed only very low amounts of amide. This group consisted of the variants Nit(Ala165Trp), Nit(Ala165Tyr), Nit(Alas165Phe), Nit(Ala165His), and Nit(Ala165Glu), and Nit(Ala165Arg). Thus, this group was identical to the group of enzyme variants that preferred (*R*)-mandelonitrile as substrate and did form only rudimentary amounts of mandelamide. This suggested that the enlargement of the amino acid residue in position 165 had a similar effect on the hydrolysis of (*R*,*S*)-2-PPN as in the hydrolysis of (*R*,*S*)-mandelonitrile and reduced for both substrates the binding or conversion of the (*S*)-enantiomers of the nitriles. The fourth group assembled those variants in which Trp188 was exchanged against a smaller amino acid residue. Surprisingly, some of these variants showed during the conversion of (*R*,*S*)-2-PPN compared to the wild-type a somehow reduced “total enantiomeric excess” for the formation of the (*S*)-enantiomers. This differed significantly from the situation found for the conversion of (*R*,*S*)-mandelonitrile (Figure 13).

## 3. Conclusions

The comparative analysis of the numerous variants generated from the nitrilase of *Pseudomonas fluorescens* EBC191 throughout the years gives an impressive example of the pronounced effects which single amino acid exchanges can have on the reaction specificity and enantioselectivity of enzymes. The mutational studies allowed the identification of two “hot spots” which determine the reaction- and enantiospecificity of the nitrilase from *P. fluorescens* EBC191 (and probably also from other organisms) to a large extent. Thus, the presence of a large aromatic residue in direct neighbourhood to the catalytical active cysteine residue (towards the C-terminus) determines nitrilases which show a pronounced reaction specificity for the formation of the acids and are highly (*R*)-selective for the formation of products such as (*R*)-mandelic acid or (*R*)-phenylglycine. There is good evidence that this substitution pattern strongly decreases the ability of the relevant (S)-nitriles to bind to the enzymes. Nitrilases with this amino acid arrangement are highly relevant for enantioselective biotransformation reactions targeting optical active α-substituted acids.

The second important amino acid residue which was identified in the course of our studies was Trp188. Homologous tryptophan residues are highly conserved among nitrilases. The exchange of this tryptophan residue against smaller amino acid residues resulted in nitrilase variants which showed a preference for the conversion of the (*S*)-enantiomers of mandelonitrile and 2-phenylpropionitrile combined with the formation of significant amounts of amides. This type of nitrilase variants might be useful for the (enantioselective) formation of α-substituted amides and could offer an alternative to true nitrile hydratases which often show limitation in respect to substrate specificity, enantioselectivity, and cyanide resistance.

In summary, our studies resulted in two catalytically very different forms of a nitrilase, which either produced from racemic mandelonitrile almost stoichiometrically the (*R*)-acids or the (*S*)-amides in a large surplus. It is tempting to speculate that these two “extremes” might indicate the limits for nitrilase-catalyzed biotransformations.

## Figures and Tables

**Table 1:** Examples for the calculation of the “total enantiomeric excess” observed during the conversion of (*R*,*S*)-mandelonitrile to mandelamide and mandelic acid.

Nitrilase	MAA (%)	ee MAA	ee MA	(*R*)-MA (%)	(*S*)-MA (%)	(*R*)-MAA (%)	(*S*)-MAA (%)	(*R*)/(*S*)	“Total Enantiomeric Excess”
Wild type	16	84 (*S*)	31 (*R*)	55	29	1	15	56/44	12 (*R*)
Del C60	52	59 (*S*)	73 (*R*)	41	7	11	41	52/48	4 (*R*)
A165H	6	1 (*R*)	91 (*R*)	90	4	3	3	93/7	86 (*R*)
W188K	94	57 (*S*)	47 (*S*)	2	4	20	74	22/78	56 (*S*)

MA: Mandelic acid, MAA: Mandelamide. The data for the amounts of mandelamide and mandelic acid formed and the enantiocomposition of the products were taken from Kiziak et al. (2007) [39], Kiziak and Stolz (2009) [46], and Sosedov and Stolz (2015) [23]. The relative concentrations of the respective enantiomers were calculated from these data.
